# Rabies virus nucleo- and glycoprotein coding sequences from dogs in Thailand (2022–2024)

**DOI:** 10.1128/mra.01268-25

**Published:** 2026-02-10

**Authors:** Lerdchai Chintapitaksakul, Kultyarat Bhakha, Peerada Siriwatcharawong, Kitipong Angsujinda, Wanchai Assavalapsakul, Nantaporn Wandee

**Affiliations:** 1 National Institute of Animal Health, Department of Livestock Development 230323, Bangkok, Thailand; 2 Aquatic Resources Research Institute, Chulalongkorn University 627646, Bangkok, Thailand; 3 Department of Microbiology, Faculty of Science, Chulalongkorn University 26683, Bangkok, Thailand; Katholieke Universiteit Leuven, Leuven, Belgium

**Keywords:** rabies virus, nucleoprotein gene, Thailand, glycoprotein gene, genomic surveillance

## Abstract

Rabies remains a major public health concern in Thailand. Nucleoprotein (N) and glycoprotein (G) genes from eight rabies-positive specimens collected during 2022 to 2024 were sequenced using Oxford Nanopore. Assemblies and phylogenetic analyses indicate genetic diversity with placement in the Asian-SEA3 lineages, supporting ongoing genomic surveillance.

## ANNOUNCEMENT

**Fig 1:**
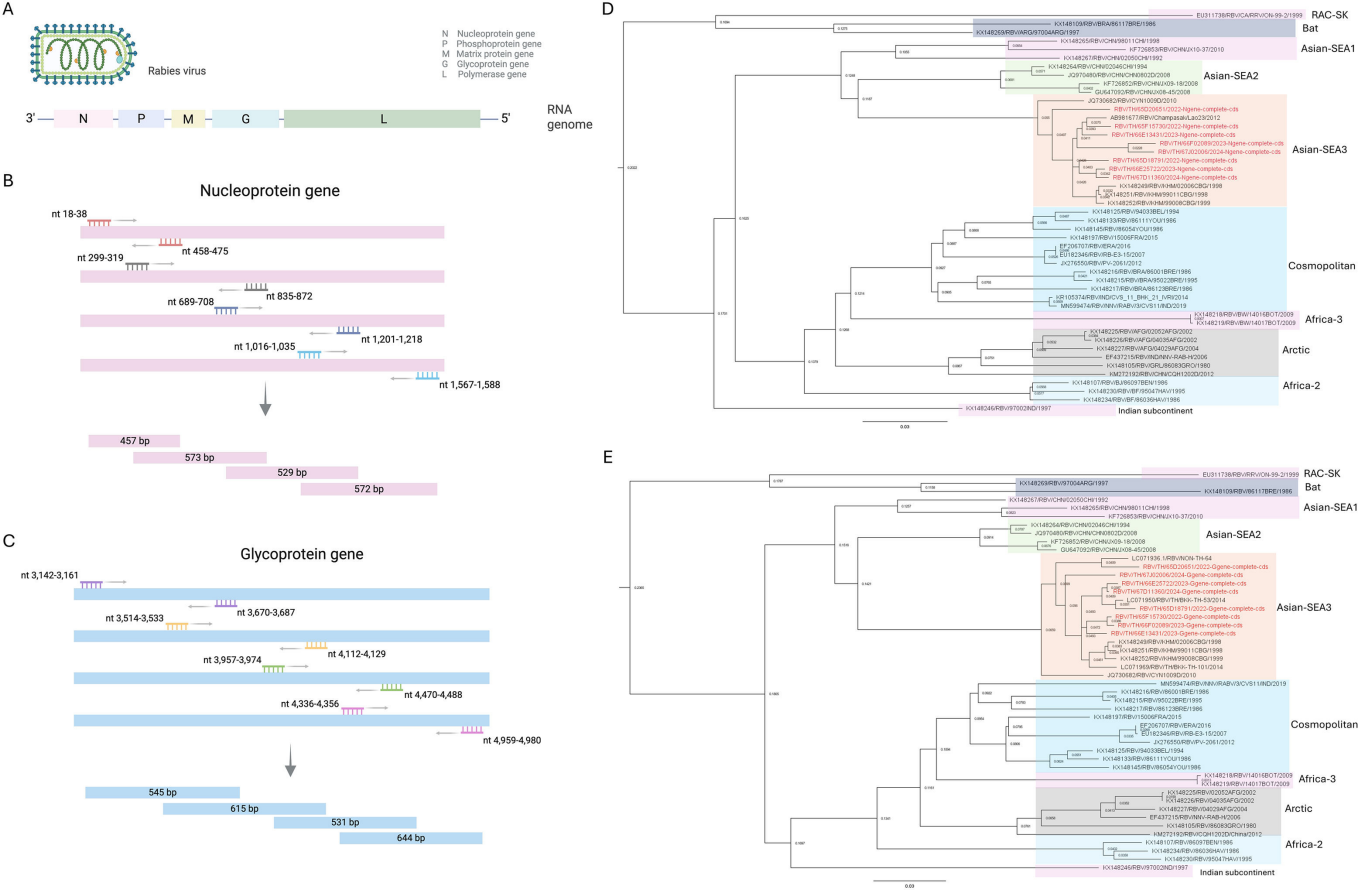
Schematic representation of (**A**) the rabies virus virion and genome organization, and the primer binding sites with overlapping RT-PCR amplicons for the (**B**) nucleoprotein and (**C**) glycoprotein genes. Nucleotide positions are numbered according to the genome of rabies virus isolate 8743THA (GenBank accession EU293121.1). Maximum-likelihood phylogenies of rabies virus (**D**) nucleoprotein and (**E**) glycoprotein coding sequences. Multiple-sequence alignments (1,353 nt for N and 1,575 nt for G) were generated using MAFFT v7 (webserver v7). Trees were inferred in IQ-TREE v1.6.12 using ModelFinder-selected nucleotide substitution models under the Bayesian Information Criterion (GTR+F+I+G4 for N; TVMe+G4 for G) and 1,000 ultrafast bootstrap replicates. Node labels indicate bootstrap support. Sequences generated in this study are shown in red in the trees. Reference sequences were retrieved from GenBank and selected according to the criteria described by Zhang et al. (2).

Rabies virus (*Rhabdoviridae*; *Lyssavirus*; Fig. 1A) remains endemic in Thailand despite control programs. Genomic surveillance of the nucleoprotein (N) and glycoprotein (G) genes supports outbreak tracing, vaccine evaluation, and elimination planning. Rabies virus phylogenies define lineages including Africa-2, Africa-3, Indian subcontinent, Arctic-related, Cosmopolitan, and Asian (1, 2). From 2016 to 2021, no genomic characterization of Thai rabies viruses appeared in public databases, despite reported outbreaks (3). This study reports N- and G-gene coding sequences from rabies-positive dog specimens to strengthen genomic surveillance and document circulating lineages. Eight rabies-positive dog brain specimens (*Canis lupus familiaris*), collected between 2022 and 2024 and confirmed by direct fluorescent antibody testing (4), were submitted from multiple Thai provinces to the National Institute of Animal Health as part of routine diagnostics. Specimens were residual diagnostic submissions and did not require further ethical approval. Samples were inactivated with TRIzol Reagent (Invitrogen), and viral RNA was extracted using the foodproof Virus RNA Extraction Kit (BIOTECON Diagnostics GmbH). N- and G-gene fragments were amplified with gene-specific primers: N-gene primers were taken from a previous report (5) and G-gene primers newly designed from rabies virus isolate 8743THA (GenBank EU293121.1). Primer binding sites and amplicon sizes are shown in Fig. 1B and C. Overlapping RT-PCR amplicons were generated using SuperScript III One-Step RT-PCR with Platinum *Taq* DNA Polymerase (Invitrogen) and 400 nM of each primer. Reverse transcription was performed at 55°C for 30 min, followed by 94°C for 2 min and 40 cycles of 94°C for 15 s, 50°C for 30 s, and 68°C for 1 min, with a final extension at 68°C for 5 min in a T100 Thermal Cycler (Bio-Rad). PCR products were purified using AMPure XP beads (Beckman Coulter).

Amplicons were barcoded using the Rapid Barcoding Kit 96 (Oxford Nanopore Technologies) and sequenced on a MinION Mk1C with an R10.4 flow cell. Basecalling used Dorado v7.6.7 in MinKNOW v24.11.8. Reads were adapter-trimmed with Porechop v0.2.4 (6), quality-assessed with NanoPlot v1.43.0 (7), and length-filtered with Chopper v0.8.0 (8), yielding 250,560 reads (141.93 Mb; read N50, 439 bp). Reads were mapped to a reference genome (GenBank ON808418.1) using minimap2 v2.28 (9), and sorted alignments and consensus sequences were generated with samtools and bcftools v1.21 (10) using default parameters. Consensus coding sequences were then combined with reference sequences from GenBank, selected according to Zhang et al. (2), and analyzed phylogenetically using MAFFT (11) and IQ-TREE (12).

Consensus assemblies recovered complete N (1,353 nt) and G (1,575 nt) coding regions. BLASTn analysis of N and G open reading frames identified the closest related strains (Table 1). Maximum-likelihood phylogenies placed all Thai sequences within the Asian-SEA3 lineage in both the N and G gene trees, with no clustering with other rabies virus lineages during the study period (Fig. 1D and E). No obvious phylogenetic incongruence suggestive of recombination was observed, although formal recombination analyses were not performed. Per-sample mean sequencing depth exceeded 100×, supporting consensus calls. Pairwise nucleotide similarity among the eight Thai sequences ranged from 95.27 to 99.60% for N and from 93.90 to 99.56% for G.

**TABLE 1:** Summary of rabies virus sequencing results and GenBank accession numbers

Gene	Sample ID	Mean sequencing depth (×)^*a*^	GC content (%)	Accession no.	Top GenBank hits (identity %, accession no.)^*b*^
N	RBV/TH/65F15730/2022	509.62	45.16	PX476146	98.60% (MN075931.1); 98.52% (AB981677.1)
	RBV/TH/65D20651/2022	194.65	45.60	PX476147	99.48% (AY218999.1); 99.33% (LC717423.1)
	RBV/TH/66E13431/2023	315.72	45.16	PX476148	99.11% (OK585051.1); 98.52% (MN075931.1)
	RBV/TH/66E25722/2023	161.07	45.31	PX476149	99.56% (PP700503.1); 99.48% (OP515296.1)
	RBV/TH/66F02089/2023	258.60	45.01	PX476150	98.45% (MW690142.1); 98.30% (MW690139.1)
	RBV/TH/67J02006/2024	118.97	45.23	PX476151	99.48% (MW690139.1); 99.48% (MW690152.1)
	RBV/TH/67D11360/2024	121.74	45.31	PX476152	99.63% (OP515296.1); 99.41% (KM366270.1)
	RBV/TH/65D18791/2022	121.74	45.23	PX476153	98.89% (ON808418.1); 98.59% (KM366242.1)
G	RBV/TH/65F15730/2022	378.15	47.43	PX476154	99.81% (LC360759.1); 99.81% (LC071983.1)
	RBV/TH/65D20651/2022	229.37	47.30	PX476155	98.10% (LC717423.1); 97.97% (AY257982.1)
	RBV/TH/66E13431/2023	314.99	47.75	PX476156	99.94% (LC360776.1); 99.87% (LC360780.1)
	RBV/TH/66E25722/2023	160.20	47.49	PX476157	99.62% (LC360769.1); 99.62% (LC360760.1)
	RBV/TH/66F02089/2023	267.39	47.24	PX476158	99.68% (LC071983.1); 99.68% (LC360759.1)
	RBV/TH/67J02006/2024	144.98	46.98	PX476159	97.20% (MG201920.1); 97.20% (GQ472552.1)
	RBV/TH/67D11360/2024	144.90	47.62	PX476160	99.87% (OP515296.1); 99.75% (LC360769.1)
	RBV/TH/65D18791/2022	137.55	47.56	PX476161	99.17% (ON808418.1); 99.11% (LC071952.1)

^*a*^ Mean sequencing depth (×) represents the average coverage of mapped reads across the consensus coding sequence of each gene.

^*b*^ Top GenBank hits indicate the first and second BLASTn matches for each sequence, reported as nucleotide identity (%), followed by the accession number; the two hits are separated by a semicolon.

## Data Availability

Sequences generated in this study have been deposited in GenBank under accession numbers PX476146–PX476161. The raw sequence data are available in the Sequence Read Archive (SRA) under BioProject PRJNA1354216 (BioSample accessions SAMN52941539–SAMN52941546).
